# LOW-INCOME, MARGINALIZED OLDER WORKERS: FINANCIAL STRAIN, HEALTH, AND MENTAL HEALTH

**DOI:** 10.1093/geroni/igad104.0888

**Published:** 2023-12-21

**Authors:** Christina Matz, Rebekah Levine Coley, Samantha Teixeira, Patrick Lai

**Affiliations:** Boston College, Boston, Massachusetts, United States; Boston College Lynch School of Education, Boston, Massachusetts, United States; Boston College School of Social Work, Boston, Massachusetts, United States; Boston College School of Social Work, Boston, Massachusetts, United States

## Abstract

Social inequalities over the life course shape later life opportunities and outcomes in important ways. Research on paid work in later life has not always captured (and has sometimes mischaracterized) the variety and complexity of lived experiences in later life—in particular for low-income workers, workers of color, women, and others marginalized due to their social position. Marginalized older workers often face greater financial instability, limited interpersonal power, and reduced access to benefits like health insurance and paid leave, all of which can lead to increased risk of early retirement, poor health/well-being, and early mortality. Trend statistics and other aggregated data often obscure how the most marginalized older adults are faring in the aging and work literature and there is a need for more research that centers these voices to inform social change, particularly in the wake of the COVID-19 pandemic. The current study aims to fill this gap by drawing on data collected in 2022/2023 among residents of a large public housing development in New England who are age 40 and older (current N=166; expected N=250). Analyses explore relationships between social resources, financial strain, health, and well-being by work status and age. Preliminary analyses suggest that certain forms of social support can buffer the negative effect of financial strain on mental health (but not physical health) among older adults living in poverty and in poor housing conditions. Implications for interventions aimed toward older marginalized adults who are working or looking for work will be discussed.

